# The associations between social environment and adolescents’ psychosomatic health: An ecological perspective

**DOI:** 10.3389/fpsyg.2023.1141206

**Published:** 2023-03-13

**Authors:** Yi Huang, Jinjin Lu, Jan Širůček

**Affiliations:** ^1^Psychology Research Institute, Faculty of Social Studies, Masaryk University, Brno, Czechia; ^2^Department of Psychology, Faculty of Social Studies, Masaryk University, Brno, Czechia; ^3^Department of Education Studies, Academy of Future Education, Xi’an Jiaotong-Liverpool University, Suzhou, China

**Keywords:** psychosomatic health, adolescent, HBSC, ecological perspective, social environment

## Abstract

**Objectives:**

It has been known that social environments are associated with adolescents’ health. However, the complex relationship between diverse types of social environments and adolescents’ psychosomatic heath remained unclear. Thus, using an ecological perspective, the current study aimed to examine the associations between social environment and adolescents’ psychosomatic health.

**Methods:**

We used the data from the Health Behavior in School-aged Children (HBSC) project conducted in the Czech Republic in 2018. A total of 13377 observations were included.

**Results:**

The region, as a macrosystem, could not explain the variance in adolescents’ psychological and somatic health. The quality of neighborhood environment (exosystem) was significantly related to adolescents’ psychological and somatic health. At the microsystem level, teacher support had stronger, family support had weaker, and peer support had no association with psychological and somatic health. At the mesosystem level, the interactions between family, teacher, and friend support were negligible for adolescents’ psychological and somatic health.

**Conclusions:**

The results underscore the importance of teachers’ support and neighborhood environment for adolescents’ psychosomatic health. Therefore, the findings suggest the need to improve teacher-adolescent relationships and the neighborhood community quality.

## Introduction

Adolescence is a turbulent life period characterized by increased emotionality, somatic and mental changes, and the increased importance of social influence (Brand and Kirov, 2011; Stormshak et al., 2011; Villalonga-Olives et al., 2011). Some scholars pointed out that during this period, adolescents are at an increased risk for subjective health complaints (Damsgaard et al., 2014). Subjective health complaints (or subjective psychosomatic complaints) refer to several common self-reported psychosomatic symptoms, including headache, backache, feeling low, sleeping difficulties, and other health indicators, by individuals with or without a medical diagnosis. However, subjective psychosomatic complaints are not causally linked with physical or psychological disease. Therefore, it is important to study the factors, such as personalities, cognitive and behavioral patterns, and environmental contributors, associated with experiencing symptoms (Eminson, 2007; Rief and Broadbent, 2007). Especially for the non-clinic population, experiencing psychosomatic symptoms has been correlated with the disadvantaged mental health condition or decreased well-being (Haugland et al., 2001; Kinnunen et al., 2010). Later studies have suggested an increasing number of adolescents have been experiencing psychosomatic symptoms (Friberg et al., 2012; Potrebny et al., 2017, 2019).

Individual factors can contribute to their psychosomatic health. First, personal demographic factors, such as gender and age, are related to adolescents’ psychosomatic symptoms. It was found that females are more likely to experience psychosomatic symptoms (Hetland et al., 2002; Potrebny et al., 2019; Inchley et al., 2020). Psychosomatic symptoms also increase with age (Petanidou et al., 2012; Potrebny et al., 2019; Inchley et al., 2020). Second, personal behavioral and psychological characteristics have been found to be associated with psychosomatic health. For example, a study indicated that tobacco use is negatively associated with adolescents’ global psychosomatic health, with self-esteem being a protective factor (Piko et al., 2016). Another research suggested that for adolescents, a positive time attitude, as one of the time cognition styles, leads to better outcomes in both psychological and somatic facets (Konowalczyk et al., 2018).

Additionally, previous studies have addressed the effects of environmental factors, ranging from family, peer relationships, school, neighborhood, and society’s perceptions, on youth’s psychosomatic health. A review work suggested the influence of family on adolescents’ health can be generally understood from demographic and psycho-socio aspects (Huang et al., 2022). For instance, many researchers have pointed out the effects of family socioeconomic status (SES) on youth’s self-reported psychosomatic symptoms, which means that disadvantaged SES is a risk factor for adolescents’ health (Kelly et al., 2010; Elgar et al., 2013; Chzhen et al., 2016). In term of family psycho-socio impacts, for instance, it was found that the good quality of parent-adolescent relationships emerges as a protective factor for adolescents’ psychosomatic problems (Hagquist, 2016). Besides, Caldwell and her colleague summarized that various parenting practices, including parent-adolescent communication, parental monitoring, and parental support, are correlated to adolescents’ health behaviors, such as substance use and early sexual initiation (Caldwell et al., 2004). Even though family is the central factor in adolescence from the developmental perspective, peer influences and school environment were also notable. The “social-brain” theory argued that the complex socio-emotional contexts impact adolescents’ social skills and cognitions later and eventually lead to certain mental health and wellbeing statuses (Wong et al., 2018). Thus, peer relationships and school experiences also strongly shape adolescents’ psychological and behavioral development. For example, for both children and adolescents, peer victimization might lead to more psychosomatic symptoms (Sumter and Baumgartner, 2017). Also, it was found that negative peer influences increase substance use, which is a health risk behavior. And in turn, substance use worsens the negative effects of peers. Contrary, peer connectedness helps to decrease substance use. It is feasible to reduce adolescents’ substance use by breaking the vicious circle between negative peer influences and substance use (McDonough et al., 2016). Regarding the influences of school settings, a cross-national study suggested that school climate strongly correlates with adolescent students’ lower frequency of self-reported psychosomatic symptoms (Freeman et al., 2012). The empirical evidence that school-based intervention is effective in promoting adolescent health also proved the significant influences of schools on adolescents’ health (Xu et al., 2020). Specifically, except for parents, teachers are significant adults for adolescents in school settings. A study suggested getting along with teachers is associated with adolescents’ better mental health (Joyce and Early, 2014). Besides family, peers, and schools which are environments that directly interact with adolescents, environments that are further away from adolescents can also have an impact on their health. For instance, the quality of neighborhood and regional differences. The good quality of the neighborhood and trust within the community also positively affected adolescents’ psychosomatic health (Åslund et al., 2010). Regional differences in adolescents’ health related outcomes are significant too. In fact, the national child-and-adolescent mental health policies differ across 30 European countries, thus causing nation-level differences in adolescents’ psychosomatic symptoms, life satisfaction, and wellbeing (Hendriks et al., 2020).

However, to our best knowledge, no study has investigated the influences of diverse environmental contexts, such as the family unit, peer relationships, school settings, and the national-level background, on adolescents’ psychosomatic health in their complex relationships. Even though a scoping review based on ecological theory pointed out the effects of various environments, like family, school, or cultural differences, on adolescents’ mental health, it was not an empirical study, and it only focused on the psychological aspect without the somatic facet (Currie and Morgan, 2020). As the ecological system theory provides a comprehensive perspective to investigate environments’ effects on youth’s health, our study adopted this theoretical framework to explain the association between social support and adolescents’ psychosomatic health while adjusting for the influence of other environmental factors, such as regional differences and the quality of the neighborhood.

The ecological system theory suggests individuals’ psychological and physical development is promoted in multilevel environments ranging from microsystem to macrosystem (Bronfenbrenner, 1979). Microsystem refers to the environments within which adolescents directly interact, including family, school, and peer relationships. Mesosystem refers to the interactions between microsystem elements, such as conversations between family and schools and parental engagement in adolescents’ peer social activities. Microsystems and mesosystem are nested within the exosystem, for example, a neighborhood, which affects adolescents indirectly. The outer layer of the ecological framework is the macrosystem, referring to the broader environments, such as regions, countries, cultural contexts, political environments, and others.

Some review studies have adopted the ecological theory to organize the past literature on adolescents’ health-related issues to synthesize the contextual determinants of adolescents’ health outcomes. For instance, Currie and Morgan (2020) reviewed the Health Behavior of School-aged Children (HBSC) database containing research from 1983 to 2020 and summarized the important influences of family, school, peer and classmate relationships, culture, country-level economy, policies, and child welfare on adolescents’ mental health. Similarly, a previous review study employed the ecological system theory to explain adolescents’ sexual risk behaviors (Kotchick et al., 2001). Moreover, the ecological theory is one of the most important guides to designing adolescents’ health-promotion programs. For instance, health education resources from family, school, health curricula, neighborhood, and community could be combined to promote adolescents’ health literacy (Higgins et al., 2009). Additionally, after tracking health promotion programs for over three decades, Wold and Mittelmark raised a whole-community approach based on the ecological theory to integrate diverse social recourses to improve adolescents’ psychological and physical functions and their healthy lifestyle (Wold and Mittelmark, 2018).

The ecological theoretical model helps synthesize the environmental effects comprehensively. Additionally, by combining the multilevel statistical approach, researchers can investigate environmental influences, for instance, by setting macrosystem-factor at a higher level in the statistical model consistent with the theoretical framework. Yet, empirical research that would explore the environmental influencers of adolescents’ psychosomatic health using the ecological framework is lacking. To expand the previous findings on adolescent psychosomatic health, our research aimed to investigate the influence of the social environment on adolescents’ psychological and somatic health from an ecological perspective. In the current study, the environments included microsystem (family, school, and peer relationships), mesosystem (the interactions between family, school, and peer relationships), exosystem (neighborhood), and macrosystem (regional context). We hypothesized that the family, teacher and friend support, and the quality of the neighborhood environment were positively related to adolescents’ psychosomatic health. Due to the limited related literature, we could not hypothesize regional differences and the interactions between family, teachers, and peers in the Czech Republic before the research.

## Materials and methods

### Data

We adopted data collected in the Czech Republic in 2018 by the Health Behavior in School-Aged Children (HBSC) survey, a cross-national survey initiated by World Health Organization.^1^

The Czech-HBSC (2017–2018) program targeted 11/13/15-year-old adolescents. To ensure the consistency of survey instruments and data collection, the process followed the standardized HBSC study protocol (Inchley et al., 2020). The Institutional Research Ethics Committee of the Faculty of Physical Culture, Palacky University, Olomouc, approved the data collection on 4 March 2016, No. 9/2016.

Schools were the primary sampling units selected randomly from the list of all eligible schools in the Czech Republic, and 227 (RR = 97%) agreed to participate in our survey. When schools agreed to participate, parents were informed about the study and asked to consent or decline their child’s participation. Adolescents themselves could also decline to participate, even if their parents approved. A team of trained administrators collected the data using an electronic questionnaire. Teachers were not present during the administration. The study included 16,065 participants, and 13,377 responses were valid (Univerzita Palackého v Olomouci, 2018).

### Measurements

#### Psychosomatic health

Eight-item *HBSC-Symptom Checklist* (HBSC-SCL) was adopted to measure adolescents’ self-reported psychosomatic health. We used three emotional-symptom items to measure *Psychological Health*, including “feeling low,” “feeling nervous,” and “feeling irritable.” The other five items measured the *Somatic Health* based on physical symptoms, including “backache,” “stomachache,” “headache,” “dizziness,” and “sleeping difficulties.” The psychological health dimension should also include “sleeping difficulties” (Gariepy et al., 2016). However, according to the Exploratory Factor Analysis (see Supplementary Table 1), the 4-factor subscale structure did not fit well the data in this study. Thus, based on EFA and other studies (Damsgaard et al., 2014; Due et al., 2019), we included the sleeping difficulties into the somatic health factor. Participants were required to rate the frequency of symptoms from 1 (“about every day”) to 5 (“rarely or never”) in the last 6 months. The instrument’s reliability in our study was acceptable (McDonald’s omega = 0.77). The McDonald’s omega values for the psychological and somatic subscales were 0.73 and 0.64, respectively.

#### Microsystem

This study considered social support from families, teachers, and friends as the microsystem factors. Family socioeconomic status (SES) was also included at this level; however, it was considered the controlled variable.

*Family Support* was measured with a four-item scale on a 7-point response options (from “very strongly disagree” to “very strongly agree”). The scale focused on four typical supportive scenarios in a family: “family tries to help the child,” “the child gets emotional support from family,” “the child talks about problems with family, “and “family is willing to help make a decision.” The McDonald’s omega value was 0.97. Because of the distribution of the mean scores of family support (see Supplementary Figure 1), we converted the mean score of family support to a three-point scale. The mean value of “1” stayed the same, and the mean value “7” was recoded as “3,” and the other mean scores were converted into “2.” The distribution of recoded scores is presented in Supplementary Figure 3.

*Teacher Support* was measured using three items on a 5-point scale (ranging from 1– “strongly agree” to 5– “strongly disagree”). The items asked about perceived acceptance by teachers, subjective feelings of care from teachers, and trust in teachers. For a clearer interpretation, we reversed the scores so that higher scores indicated more support from teachers. The McDonald’s omega value was 0.82.

*Friends’ Support* was measured with a 4-item scale on a 7-point scale (1 – “very strongly disagree” to 7 – “very strongly agree”). The items asked about the friends’ help, reliability, sharing, and problem talking. The McDonald’s omega value was 0.94. Due to the distribution of friend support’s mean scores (see Supplementary Figure 2), we converted them to a three-point scale using the same method as for family support (see Supplementary Figure 4).

*The Family Affluence Scale* (FAS) measured socioeconomic status. The scale included six items that aimed to investigate the family material affluence, including the number of cars, bathrooms, computers, and bathrooms; having a dishwasher at home; the frequency of family holidays; and adolescents having their own bathroom. FAS was proved as an effective indicator of SES (Hobza et al., 2017).

#### Mesosystem

We defined three new variables of “family support * teacher support,” “family support * friend support, “and “teacher support * friend support” to present the interactions between family, teachers, and peer relationships. Some literature focused on applying the ecological model to children’s health promotion has suggested this statistical moderation approach to represent the mesosystem (McIntosh et al., 2008; Gubbels et al., 2014). Notably, the interactions indicated whether the effect of one variable depended on another variable. For instance, “family support * teachers’ support” measured whether the teachers’ support and family support magnified or demoted each other’s effect on adolescents’ psychosomatic health.

#### Exosystem

*The Quality of the Neighborhood* was measured with a six-item scale. Participants were required to rate their subjective feelings toward the neighborhood (e.g., “it is good to live in the area”) and their experiences (e.g., “you can trust people around”) on a scale ranging from 1 (“agree a lot”) to 5 (“disagree a lot”). We reverse-coded the scores, with a higher score indicating a better neighborhood environment. The reliability was acceptable (McDonald’s omega = 0.78).

#### Macrosystem

In this study, the macrosystem-level variable was the region where adolescents lived during the survey time. The Czech Republic has 14 regions. Related evidence showed regional economic inequalities in the Czech Republic, such as gross domestic product, net disposable income, incomes from the perspective of the structure, personal income taxes, and health and social insurance (Skaličková et al., 2014).

### Data analysis

SPSS 25.0 was used to conduct statistical analysis. First, we conducted descriptive statistics to describe the characteristics of our sample. Second, we ran the Pearson correlation analysis between independent variables and psychosomatic health. Subsequently, we built the multilevel regression model. According to the ecological theory, in our multi-regression model, we set family support, teachers’ support, friends’ support, interactions between the three supports, and neighborhood environment at level 1, where gender, grade, and family SES background were control variables. The region belonged to level 2. If the variance explained by regional difference were lower than 1%, suggesting that the regional difference did not significantly contribute to adolescents’ psychosomatic health (Bliese, 1998), we continued to compute a general linear model (GLM), setting all the variables except region at the same level.

## Results

### Descriptive results

The descriptive statistics are shown in Table 1.

**Table 1:** Descriptive statistics for sample characteristics and the Chi-square test between independent factors and adolescents’ psychological/somatic health.

		N (%)	Mean	S.D.
Dependent variable	Psychological health	12,811	3.52	1.03
	Somatic health	12,793	4.23	0.71

Individual-level	Sex			
	Male	6,808(50.9%)		
	Female	6,569(49.1%)		
	Grade			
	11-year-old grade	4,380(32.7%)		
	13-year-old grade	4,654(34.8%)		
	15-year-old grade	4,343(32.5%)		
Microsystem	Family SES	13,335	2.34	0.40
	Teachers’ support	13,099	3.57	0.89
	Family support	12,544	2.20	0.65
	Friends’ support	12,981	2.09	0.51
Exosystem	Neighborhood Environment	11,915	3.83	0.74
Macrosystem	Region			
	Praha	931(7.0%)		
	Stredocesky	1,125(8.4%)		
	Jihocesky	1,017(7.6%)		
	Zapadocesky	1,043(7.8%)		
	Karlovarsky	924(6.9%)		
	Ustecky	897(6.7%)		
	Liberecky	821(6.1%)		
	Kralovehradecky	869(6.5%)		
	Pardubicky	997(7.5%)		
	Vysocina	852(6.4%)		
	Jihomoravsky	844(6.3%)		
	Olomoucky	955(7.1%)		
	Zlinsky	960(7.2%)		
	Moravskoslezsky	1,142(8.5%)		

According to the Pearson correlation analysis, all social supports and the quality of the neighborhood environment were positively correlated with psycho-health (see Table 2). However, the effect size of the correlation between friends’ support and psychological health was close to 0. Regarding adolescents’ somatic health, the effect sizes of teacher support and neighborhood environment were higher. The effect size of the correlation between family support and somatic health was very small. The association between friend support and somatic health was non-significant.

**Table 2:** Correlations between social support, neighborhood environment, and adolescents’ psychosomatic health based on the average score of each scale.

	1	2	3	4	5
					
1. Psycho-health	–				
2. Somatic-health	0.55^**^	–			
3. Friends’ support	0.03^**^	0.01	–		
4. Teachers’ support	0.22^**^	0.20^**^	0.00	–	
5. Family support	0.10^**^	0.07^**^	0.26^**^	0.08^**^	–
6. Neighborhood environment	0.21^**^	0.19^**^	0.02^*^	0.28^**^	0.06^**^

^**^p < 0.01, ^*^p < 0.05.

Next, we built a multilevel regression model based on each variable’s standardized, average score. We entered the region at level 2 and other predictors at level 1. Intraclass Correlation (ICC) coefficients were 0.004 and 0.002 for the psychological-health-focused and somatic-health-focused models, respectively. The results indicated no regional differences in adolescents’ psychological and somatic health. Therefore, we computed two GLM models in the following step. The first model included all predictors in the microsystem and exosystem. In the second model, we also entered the mesosystem predictors, the interactions between family, friends, and teachers’ support. The purpose of constructing two models was to avoid the co-linear relationship between microsystem and mesosystem. To better interpret the mesosystem’s effect, we compared the two models’ R-square values.

According to the first GLM model results based on standardized scores, teacher support and the quality of the neighborhood environment had significantly greater positive effects on psycho-health compared to family support (see Table 3). The association between friend support and psychological health was not significant. This model accounted for 11.2% of the variance in adolescents’ psychological health. After adding the interactions of three social supports, the model improved the variance explained by less than 1% (R-square = 11.4%), suggesting negligible effects of interactions between family, teacher, and friend support. According to the coefficients, the effect sizes of these interactions were near zero (see Table 4).

**Table 3:** The GLM model investigating the effects of three social supports on adolescents’ psychological/somatic health based on the ecological theory, excluding the mesosystem.

Dependent variable		Beta	Std. Error	Sig.	Lower Bound	Upper Bound
Psychological health	Intercept	−0.212	0.018	0.000	−0.247	−0.177
	Sex (boy vs. girl)	0.322	0.018	0.000	0.287	0.358
	Grade (11- year vs. 15-year)	0.185	0.023	0.000	0.139	0.231
	Grade (13-year vs. 15 year)	−0.024	0.021	0.262	−0.066	0.018
	SES	0.021	0.009	0.019	0.003	0.039
	SFam	0.072	0.009	0.000	0.054	0.091
	STea	0.154	0.010	0.000	0.135	0.173
	SFri	0.014	0.009	0.122	−0.004	0.033
	Neighborhood	0.131	0.009	0.000	0.113	0.150
Somatic Health	Intercept	−0.192	0.018	0.000	−0.227	−0.156
	Sex (boy vs. girl)	0.320	0.018	0.000	0.285	0.356
	Grade (11- year vs. 15-year)	0.082	0.024	0.001	0.036	0.129
	Grade (13-year vs. 15 year)	0.023	0.022	0.283	−0.019	0.065
	SES	−0.001	0.009	0.878	−0.019	0.017
	SFam	0.042	0.009	0.000	0.024	0.061
	STea	0.145	0.010	0.000	0.126	0.164
	SFri	0.004	0.009	0.704	−0.015	0.022
	Neighborhood	0.131	0.010	0.000	0.112	0.150

SFam referred to support from family. STea referred to support from teachers, and SFri referred to support from friends.

**Table 4:** The complete ecological model using a multilevel regression to investigate adolescents’ psychological/somatic health.

Dependent variable		Beta	Std. Error	Sig.	Lower Bound	Upper Bound
Psychological health	Intercept	−0.217	0.018	0.000	−0.252	−0.182
	Sex (boy vs. girl)	0.325	0.018	0.000	0.290	0.360
	Grade (11- year vs. 15-year)	0.184	0.023	0.000	0.138	0.230
	Grade (13-year vs. 15 year)	−0.023	0.021	0.280	−0.065	0.019
	SES	0.020	0.009	0.026	0.002	0.038
	SFam	0.080	0.010	0.000	0.061	0.099
	STea	0.151	0.010	0.000	0.132	0.170
	SFri	0.015	0.009	0.101	−0.003	0.034
	Neighborhood	0.129	0.010	0.000	0.110	0.148
	SFam * SFri	0.023	0.008	0.003	0.008	0.038
	SFam * STea	−0.024	0.009	0.009	−0.042	−0.006
	STea * SFri	0.000	0.008	0.978	−0.017	0.016
Somatic health	Intercept	−0.193	0.018	0.000	−0.229	−0.158
	Sex (boy vs. girl)	0.321	0.018	0.000	0.286	0.357
	Grade (11- year vs. 15-year)	0.082	0.024	0.001	0.036	0.129
	Grade (13-year vs. 15 year)	0.024	0.022	0.270	−0.018	0.066
	SES	−0.002	0.009	0.821	−0.020	0.016
	SFam	0.047	0.010	0.000	0.028	0.066
	STea	0.143	0.010	0.000	0.124	0.162
	SFri	0.006	0.009	0.558	−0.013	0.024
	Neighborhood	0.130	0.010	0.000	0.111	0.149
	SFam * SFri	0.009	0.008	0.271	−0.007	0.024
	SFam * STea	−0.016	0.009	0.085	−0.035	0.002
	STea * SFri	−0.008	0.009	0.373	−0.024	0.009

SFam referred to support from family. STea referred to support from teachers, and SFri referred to support from friends.

The region explained only 0.2% of the variance in adolescents’ somatic health. Other social supports, except for the friend support, had statistically significant positive effects on adolescents’ somatic health. However, the effect size of family support was very small (see Table 3). The first model that excluded the interactions between family, teacher, and friend support explained 8.8% of the variance in somatic health. In the second model that included social support interactions, the variance explained remained the same (R-square = 0.088). The unchanged R-square value suggested the unobserved effect of interactions of the three social supports on adolescents’ somatic health. Moreover, the interactions between the three social supports were not significant (see Table 4). The neighborhood environment had a significantly positive correlation with somatic health.

## Discussion

This study examined mainly the effects of social environment on adolescents’ psychosomatic symptoms from the ecological perspective, which considers the effects of different environments on youth’s development. First, according to the multilevel model, this study did not find an obvious association between region as the macrosystem element and adolescents’ self-reported psychosomatic health in the Czech Republic. The GLM model suggested that the quality of the neighborhood environment, which belongs to the exosystem, influenced both psychological and somatic health positively and significantly. Finally, the results showed that at the microsystem level, among the three social supports, teacher support was the most significant protective factor for adolescents’ psychological health, followed by family support. The effect of friend support was non-significant for mental health. Teacher support had a significantly positive effect on somatic health, while the effect of family support was minimal. Friend support did not correlate with adolescents’ somatic health. In addition, the interactions between three social supports (belonging mesosystem) did not influence psychological and somatic symptoms.

The most important finding of the current study is that support from teachers and family decreases the risk of adolescents’ self-reported psychological health problems. Compared to family support, teacher support had a greater protective effect. These findings are consistent with prior studies. First, driven by the ecological theory, teachers provide the support that helps adolescents achieve more positive outcomes. A systematic review pointed out that harmony and supportive teacher-student relationships reduce adolescents’ problematic behaviors and psychological symptoms (Schulte-Körne, 2016). Second, adolescents interact frequently with their families, which also provide support and are a very meaningful protective factor for adolescents’ mental health (McConnell et al., 2015, 2016). Third, the finding that family support is a weaker predictor compared to teacher support is consistent with a previous meta-analysis (Chu et al., 2010). On the other hand, according to some classical theories, family is also a source of conflict, which may lead to negative outcomes for adolescents (Rook, 1984; Barrera et al., 1993).

According to our findings, family and teacher support influences on adolescents’ somatic health are similar, although teacher support was a stronger predictor of somatic health. The weak influence of family support on adolescents’ somatic health may be due to family members’ lack of related somatic health awareness. Previous studies have suggested that for physiological health problems, the direct support of health behaviors and health management is more important for decreasing somatic symptoms compared to only emotional support (Lorig and Holman, 2003; Williams et al., 2006). However, direct health support is based on the knowledge or experiences of health management strategies (Williams et al., 2006; Leroy et al., 2017). This finding suggests the possible limited physiological knowledge of the general public in the Czech Republic. Families must master essential health knowledge to promote children and adolescents’ psychological and physical development. Thus, there is a need for public health education for Czech citizens.

Regarding the positive effect of teacher support on adolescents’ somatic health, our results reflected the significant achievement of the health education program established in 1999. Advocated by the Health Literacy Portal since 2006, the health education program has focused on the Urgent First Aid and Safe Behavior and Prevention of Infectious Disease in the Czech Republic (Health Literacy Portal, 2006; Reissmannová, 2021). Our results demonstrated that Czech teachers’ support promotes adolescents’ physical development to some extent.

This study indicated that the effect of friend support on psychological and somatic health in Czech is negligible, which is in line with some prior studies. For example, peer support did not affect Slovak adolescents’ excessive Internet use, which is a problematic behavior highly correlated with mental health (Blinka et al., 2020). According to a meta-analysis, peer support has much less influence on adolescents’ well-being compared to teacher and parent support (Chu et al., 2010). The reason might be that, for youth, peer support is the only social support resource that they choose autonomously. Thus, youth tend to get close to someone sharing similar characteristics (Erdley et al., 2001). From this perspective, the influences of peer relationships may not be obvious.

The effects of three social supports on adolescents’ psychosomatic health are independent. In this study, teacher support, family support, and peer support did not magnify or compensate for each other’s effects. First, the interaction between teachers and family was weak in the Czech Republic, which means teacher support does not enhance the positive effect of family support. This result is not surprising, as communication between family and school is lacking in the Czech Republic. A previous study highlighted that the collaboration between school and family had been an unresolved issue, which, like matched interventions, has been getting limited attention in the Czech Republic (Dusi, 2012). Many western countries, such as the United Kingdom and the United States, have been engaged in diverse programs to promote efficient communication between teachers and parents regarding children’s and adolescents’ academic performance and well-being (Thompson et al., 2018). The gap between Czech and other developed countries has revealed the necessity to design relevant programs to enhance school-family cooperation for the next generation.

Similarly, the current study pointed out the inadequate family-peer and teacher-peer interactions, which could be due to the lack of communication between school and family. As a result, families and teachers cannot understand adolescents’ peer relationships in other contexts. For example, because parents do not often communicate with teachers, they do not know about adolescents’ social activities with peers in school, making monitoring adolescents’ school-social-network more difficult. Likewise, teachers cannot get to know adolescents’ social networks in their communities as parents rarely communicate with them. Because of insufficient engagement in adolescents’ peer relationships, intervening in risky adolescents’ peer relationships becomes problematic.

Except for the influence of three types of social support, this study also suggested the important effect of the neighborhood on adolescents’ psychosomatic health. Mmari and his colleagues (2014) summarized the neighborhood as one of the critical social environments that influence adolescents’ health and safety from two perspectives, social capital and social cohesion. Social capital focuses on interpersonal relationships, and social cohesion emphasizes the degree to which neighbors share instrumental and emotional support. Both factors were previously associated with adolescents’ health outcomes. For instance, it was found that social cohesion correlates with adolescents’ health behaviors (Mmari et al., 2014) and mental health (Hurd et al., 2013). Besides, as one aspect of social capital, trustful and helpful neighborhood relationship reduces youth’s psychological health problems (Chung and Docherty, 2011). A systematic review synthesizing global evidence showed supporting underprivileged neighborhoods effectively reduced adolescents’ health risk (Sellström and Bremberg, 2006). Therefore, based on previous successful experiences and the current results, scholars have called for the related interventions to improve neighborhood community environment and subsequently adolescents’ psychological and somatic health in the Czech Republic.

Finally, our results demonstrated minimal regional differences in adolescents’ psychosomatic health. Regional differences accounted only for 0.4 and 0.2% of the variance in psychological and somatic health, respectively, in the current sample. This finding may be explained by the health equality in the Czech Republic. Europe-wide Report suggested that according to the Gini coefficient ranking, which is an income inequality index, Czech was the third-highest country among OECD members in 2018 in terms of health equality (Lánský and Tomková, 2018). Moreover, Czech provides universal-coverage health insurance for its citizens, which means there are no financial difficulties in seeking necessary physical and psychiatric medical help (Lánský and Tomková, 2018). From this perspective, the health inequality crossing regions in the Czech Republic should not be obvious.

This paper first addressed the effects of social support on adolescents’ psychosomatic health from the comprehensive ecological framework in the Czech Republic. However, it had some limitations. First, we used only sectional data. A longitudinal design is necessary to establish causal relationships. Researchers may consider collecting longitudinal data to construct the cross-lagged model in the future. Second, the adolescents completed self-reported questionnaires, which meant subjective bias might exist in the study. A previous study has indicated that teachers encourage parental support and involvement (Williams et al., 2007). Parental and adolescents’ perceptions of supportive or impaired communications may differ (Yu et al., 2006). Thus, we suggest further studies investigating this issue from the parents’ or teachers’ perspectives. Third, the internal consistency of the somatic health measurement was not high (McDonald’s omega = 0.64) in our sample, which means the scale’s psychometric properties should be evaluated in the Czech adolescent sample. In fact, in 2001, a study across four European countries already pointed out the possible problematic re-test reliability of somatic symptoms measurement (Haugland et al., 2001), suggesting some additional somatic symptoms may be considered when measuring adolescents’ somatic health, such as neckache and general body ache (Haugland and Wold, 2001). Thus, the limited number of somatic-symptom items might also be the reason for the unsatisfactory reliability of the somatic health subscale. According to the unsatisfied result of the exploratory factor analysis for the symptom checklist, we recommend future researchers use the exploratory structural equation method to examine the psychometric properties of the measurement. Moreover, we advise using the latent estimation of the psychosomatic health based on the observed symptoms for further analysis, because the psychometric model construction is possibly complex, for instance, sleeping difficulties may contribute factor loadings to psychological health and somatic health at the same time.

The current study suggests the implication of school and community based psychological interventions for adolescents’ psychosomatic health. According to our findings that teachers play an important role in Czech adolescents’ psychosomatic health, it is necessary to establish a school counseling system to help teachers to monitor adolescents’ psychosomatic development and cooperate with school psychologists to intervene in adolescents’ adverse development. Also, considering the influence of the community on adolescents, combing professional social workers to provide psychological support resources to communities is meaningful in the Czech Republic. Finally, based on the preceding experiences in other countries, we suggest related psychological intervention programmes to strengthen the cooperation between parents and schools.

## Data availability statement

The datasets presented in this study can be found in online repositories: https://hbsc.org/data/.

## Ethics statement

This study on human participants was reviewed and approved by The Institutional Research Ethics Committee of the Faculty of Physical Culture, Palacky University, Olomouc on 4 March 2016, No. 9/2016. Written informed consent to participate in this study was provided by the participants’ legal guardian/next of kin.

## Author contributions

YH conceptualized the idea, and contributed to study design, data analysis, and the first draft of the entire article. JS in participated the data analysis. JL reviewed and re-edited articles. JS and JL approved the final edited version and approved the submitted version. All authors reviewed and approved the final edited version and approved the submitted version.

## Funding

This study was supported by the Masaryk University (research grant MUNI/A/1554/2021) and the Czech Science Foundation (research grant GA19-22997S).

## Conflict of interest

The authors declare that the research was conducted in the absence of any commercial or financial relationships that could be construed as a potential conflict of interest.

## Publisher’s note

All claims expressed in this article are solely those of the authors and do not necessarily represent those of their affiliated organizations, or those of the publisher, the editors and the reviewers. Any product that may be evaluated in this article, or claim that may be made by its manufacturer, is not guaranteed or endorsed by the publisher.

## Footnotes

^1^www.hbsc.org
