# Supplementary material for: The associations between social environment and adolescents’ psychosomatic health: An ecological perspective
Source: Front Psychol. 2023 Mar 13;14:1141206. doi: 10.3389/fpsyg.2023.1141206 (PMC10040858; doi:10.3389/fpsyg.2023.1141206)
Supplement: Supplementary file 1 [file Table_1.DOCX]

**Supplementary Materials**

**Figure 1. The distribution of mean scores of family support**


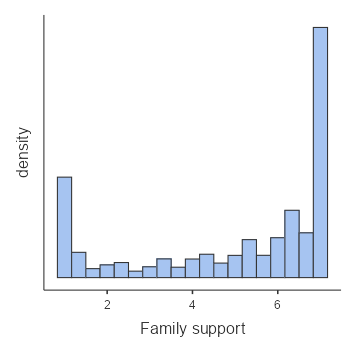


**Figure 2. The distribution of mean scores of friend support**


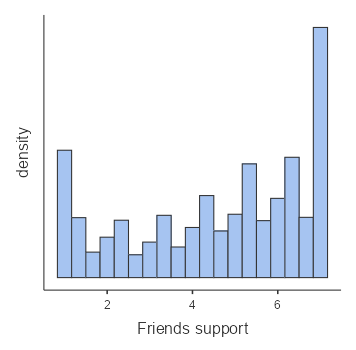


**Figure 3. The distribution of mean scores of family support after recoding**


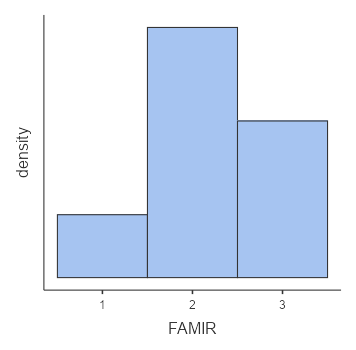


Family support

**Figure 4. The distribution of mean scores of friend support after recoding**


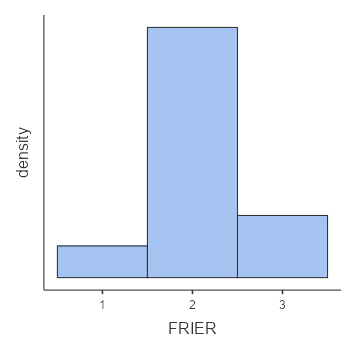


Friends' support

**Table 1. Exploratory Factor Analysis of the HBSC-symptoms Checklist**

|  | | | | | | | |
| --- | --- | --- | --- | --- | --- | --- | --- |
|  | | **Factor Loadings** | | | |  | |
|  | | **1** | | **2** | | **Uniqueness** | |
| Irritable |  | 0.81 |  |  |  | 0.40 |  |
| Feeling low |  | 0.63 |  |  |  | 0.52 |  |
| Nervous |  | 0.51 |  |  |  | 0.65 |  |
| Headache |  |  |  | 0.60 |  | 0.62 |  |
| Dizzy |  |  |  | 0.55 |  | 0.73 |  |
| Stomachache |  |  |  | 0.52 |  | 0.73 |  |
| Backache |  |  |  | 0.36 |  | 0.82 |  |
| Sleeping difficulties |  |  |  | 0.30 |  | 0.75 |  |
| Note. 'Maximum likelihood' extraction method was used in combination with an 'oblimin' rotation (hiding loadings below 0.30) | | | | | | | |
|  | | | | | | | |
